# Blood free-circulating DNA testing by highly sensitive methylation assay to diagnose colorectal neoplasias

**DOI:** 10.18632/oncotarget.24768

**Published:** 2018-03-30

**Authors:** Yutaka Suehiro, Shinichi Hashimoto, Shingo Higaki, Ikuei Fujii, Chieko Suzuki, Tomomi Hoshida, Toshihiko Matsumoto, Yuko Yamaoka, Taro Takami, Isao Sakaida, Takahiro Yamasaki

**Affiliations:** ^1^ Department of Oncology and Laboratory Medicine, Yamaguchi University Graduate School of Medicine, Ube, Japan; ^2^ Department of Gastroenterology and Hepatology, Yamaguchi University Graduate School of Medicine, Ube, Japan; ^3^ Department of Gastroenterology, Sentohiru Hospital, Ube, Japan; ^4^ Ajisu Kyoritsu Hospital, Yamaguchi, Japan

**Keywords:** colorectal cancer, droplet digital PCR, hTERT, liquid biopsy, methylated TWIST1

## Abstract

Although methylated TWIST1 is a biomarker of colorectal neoplasia, its detection from serum samples is very difficult by conventional bisulfite-based methylation assays. Therefore, we have developed a new methylation assay that enables counting of even one copy of a methylated gene in a small DNA sample amount without DNA bisulfite treatment. We performed this study to evaluate the sensitivity and specificity of serum DNA testing by the new methylation assay in combination with and without the fecal immunochemical test for hemoglobin for the detection of colorectal neoplasia. This study comprised 113 patients with colorectal neoplasia and 25 control individuals. For the new methylation assay, DNA was treated in two stages with methylation-sensitive restriction enzymes, followed by measurement of copy numbers of hTERT and methylated TWIST1 by multiplex droplet digital PCR. The fecal immunochemical test had a sensitivity of 8.0% for non-advanced adenoma, 24.3% for advanced adenoma, and 44.4% for colorectal cancer, and a specificity of 88.0%. The new assay had a sensitivity of 36.0% for non-advanced adenoma, 30.0% for advanced adenoma, and 44.4% for colorectal cancer, and a specificity of 92.0%. Combination of the both tests increased the sensitivity to 40.0%, 45.7%, and 72.2% for the detection of non-advanced adenoma, advanced adenoma, and colorectal cancer, respectively, and resulted in a specificity of 84.0%. Combination of both tests may provide an alternative screening strategy for colorectal neoplasia including potentially precancerous lesions and colorectal cancer.

## INTRODUCTION

Colorectal cancer (CRC) is the second most commonly diagnosed cancer in females and the third most in males in the world [1]. It is estimated that 1.4 million new cases and 693,900 deaths occurred worldwide in 2012 [1]. Because more than 95% of patients with CRC would benefit from curative surgery if diagnosed at an earlier or precancerous stage [2], it is important to develop highly sensitive and specific assays to detect precancerous lesions and CRC at the early stage that are non-invasive, inexpensive, and easy to perform.

The main approach to CRC screening throughout the world is the fecal immunochemical test for hemoglobin (FIT), and patients with fecal hemoglobin >20 μg hemoglobin/g feces (equivalent to a 100 ng/mL cutoff of hemoglobin in sample buffer) are referred for colonoscopy [3]. Although the sensitivity of the FIT for the diagnosis of colorectal neoplasia is 73.8% for CRC, it falls to 65.5% for the detection of Stage I CRC and to 23.8% for the detection of potentially advanced precancerous lesions [4]. Furthermore, the FIT also carries the risk of false-positive results in patients with hemorrhoids, ulcers, and inflammatory bowel disease [5–7]. To avoid the disadvantages of the FIT, more sensitive and specific screening methods are required. One solution is the free-circulating methylated DNA test in blood. In 2016, the U. S. Food and Drug Administration approved Epi proColon (Epigenomics AG, Berlin, Germany), the first blood-based colorectal screening test consisting of DNA testing of methylated SEPT9 [8]. However, blood-based DNA tests including Epi proColon require a large amount of serum or plasma sample (~3.5 mL) [8] because the content of cancer-specific DNA in blood (serum or plasma) is very small [9, 10] and conventional DNA methylation assays require bisulfite treatment of DNA, which causes degradation and loss of DNA [11, 12]. Because a large-scale experiment is time-consuming and expensive, to overcome these problems, we have developed a new assay called the combined restriction digital PCR (CORD) assay, which enables counting of even one copy of a methylated gene in a small DNA sample amount without DNA bisulfite treatment [13]. In addition, as we were the first in the world to report that methylated TWIST1 is a biomarker of colorectal neoplasias [14], we evaluated the clinical performance of the serum CORD assay, targeting methylated TWIST1 in combination with and without FIT for the detection of colorectal neoplasia from serum samples, and compared clinical performance between TWIST1 and SEPT9 (a marker of the Epi proColon).

## RESULTS

### FIT

**Figure 1:**
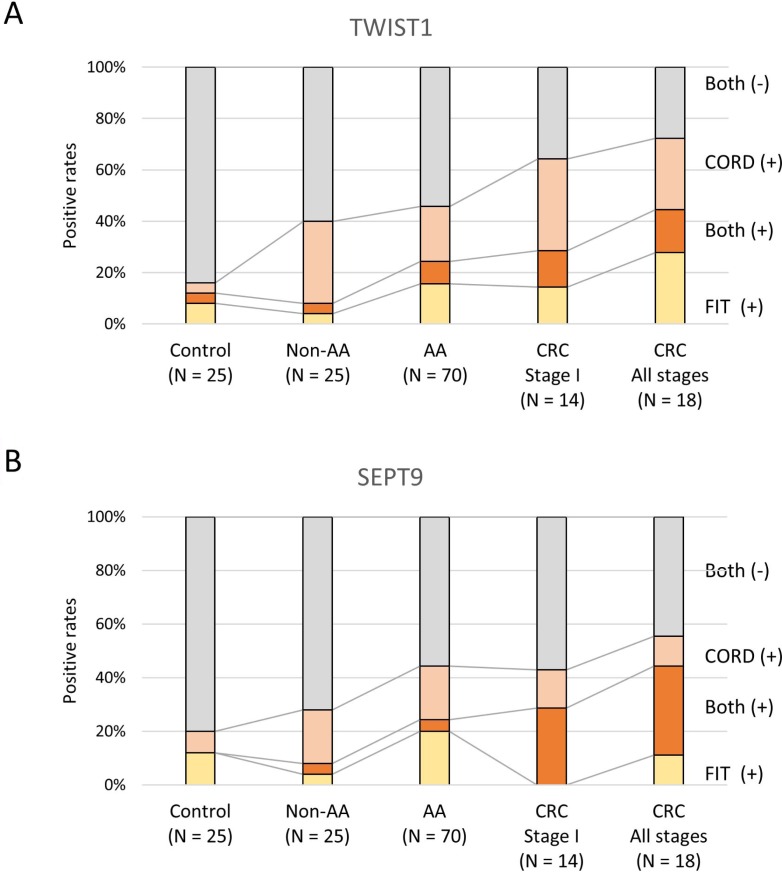
Distribution of the results of FIT and serum CORD assays of TWIST1 (**A**) and SEPT9 (**B**). Both (+): both FIT and serum CORD assay are positive; Both (−): both FIT and serum CORD assay are negative; CORD: serum combined restriction digital PCR assay; FIT: fecal immunochemical test.

FIT resulted in a sensitivity of 8.0% (2/25) for non-advanced adenoma, 24.3% (17/70) for advanced adenoma, and 44.4% (8/18) for CRC screening, with a specificity of 88.0% (22/25) (Table 1 and Figure 1).

**Table 1:** Sensitivity and specificity of FIT, CORD assay, and the combination of FIT and CORD assay for the findings of colonoscopy

Most advanced findings	Colonoscopy (*N* = 138)	FIT (*N* = 138)				Serum CORD assay of TWIST1 (*N* = 138)				Combination (*N* = 138)			
		Positive results	Specificity	*P* value	OR (95% CI)	Positive results^a^	Specificity	*P* value	OR (95% CI)	Positive results^b^	Specificity	*P* value	OR (95% CI)
	**No.**	**No.**	**%**			**No.**	**%**			**No.**	**%**		
Negative results on colonoscopy	25	3	88.0		1.000 (Reference)	2	92.0		1.000 (Reference)	4	84.0		1.000 (Reference)
			**Sensitivity** **(95% CI)**				**Sensitivity** **(95% CI)**				**Sensitivity** **(95% CI)**		
Non-advanced adenoma	25	2	8.0 (1.0–26.0)	1.0000	0.6377 (0.09704–4.190)	9	36.0 (18.0–57.5)	0.0374	6.469 (1.230–34.026)	10	40.0 (22.1–61.3)	0.1137	3.500 (0.9203–13.311)
Advanced adenoma	70	17	24.3 (14.8–36.1)	0.2594	2.352 (0.6255–8.845)	21	30.0 (19.6–42.1)	0.0306	4.929 (1.064–22.830)	32	45.7 (33.7–58.1)	0.0089	4.421 (1.374–14.221)
Colorectal cancer (stage I)	14	4	28.6 (8.4–58.1)	0.2251	2.933 (0.5502–15.639)	7	50.0 (23.0–77.0)	0.0052	11.500 (1.929–68.548)	9	64.3 (35.1–87.2)	0.0041	9.450 (2.047–43.263)
Colorectal cancer (all stages)	18	8	44.4 (21.5–69.3)	0.0312	5.867 (1.279–26.914)	8	44.4 (21.5–69.3)	0.0092	9.200 (1.650–51.305)	13	72.2 (46.6–90.3)	0.0004	13.650 (3.089–60.324)

95% CI: 95% confidence interval; CORD: combined restriction digital PCR; FIT: fecal immunochemical test for hemoglobin; OR: odds ratio.

^a^Criterion for a positive result of the CORD assay is the presence of 2.8 or more copy numbers of methylated TWIST1.

^b^Criterion for a positive result with the combination of FIT and CORD assay is either a positive FIT or CORD assay or both are positive.

### Carcinoembryonic antigen (CEA)

Increased CEA (≥6 ng/mL) was found in 1/6 (16.7%) patients in the non-advanced adenoma group, 9/54 (16.7%) in the advanced adenoma group, and 17.6% (3/17) in the CRC group.

### Basic performance test of CORD assay

**Figure 2:**
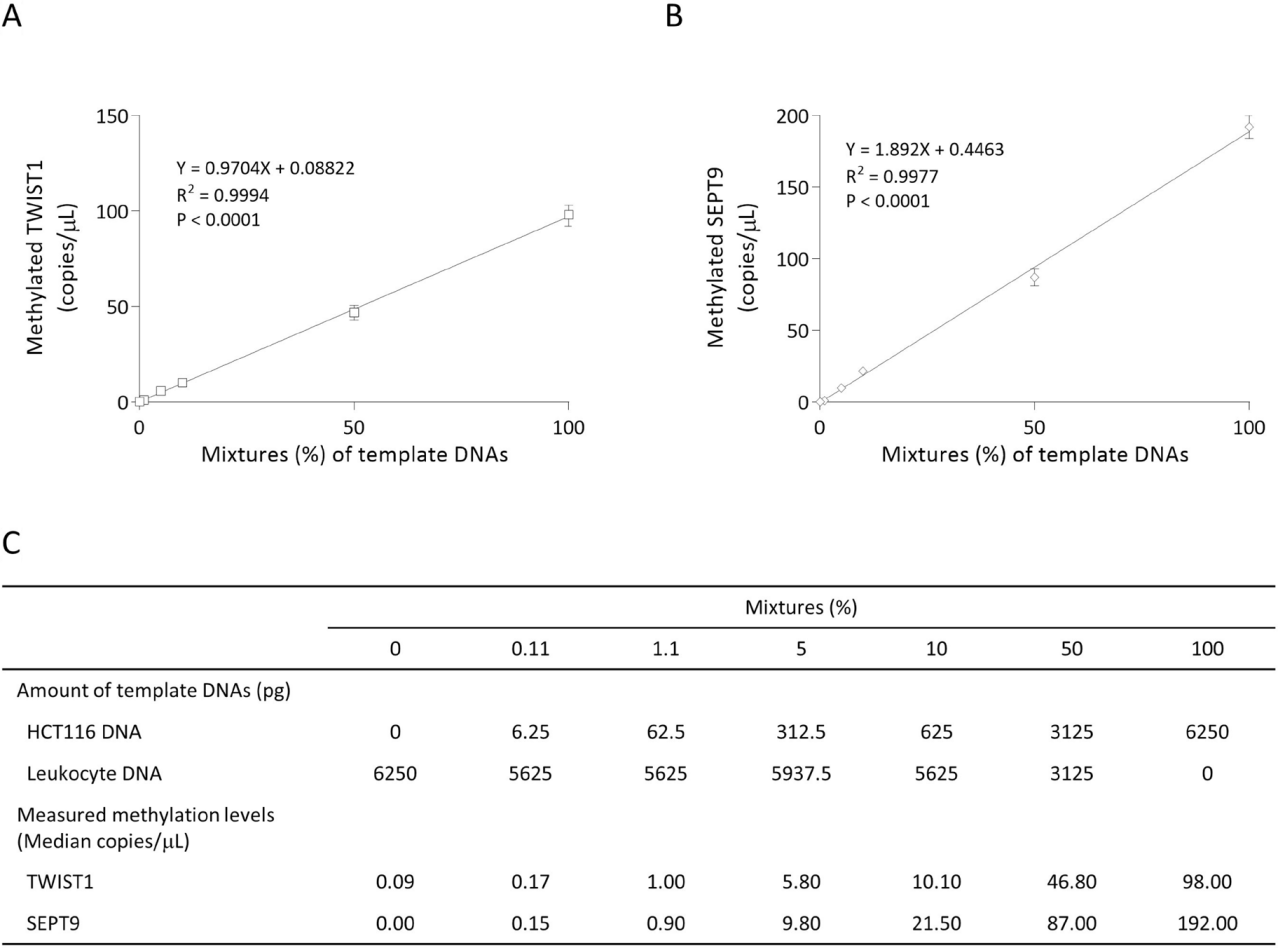
Basic performance tests of CORD assay Percentages on the x-axis indicate the ratios of HCT116 DNA to leukocyte DNA in the template DNA. Experimentally determined methylated copy numbers for TWIST1 (**A**) and methylated SEPT9 (**B**) are shown on the Y-axis, and these data are summarized in (**C**). The error bars represent Poisson 95% confidence intervals.

For the basic performance test of the CORD assay to detect hypermethylated cancer-derived DNA against a background of blood-derived DNA, we spiked DNA from colon cancer cell line HCT116 (control DNA for methylation of TWIST1 and SEPT9) at ratios of 100%, 50%, 10%, 5%, 1.1%, 0.11%, and 0% into DNA extracted from leukocyte DNA (control DNA for unmethylation of TWIST1 and SEPT9) and measured the methylation levels of TWIST1 and SEPT9 for each sample. As shown in Figure 2A–2C, the CORD assay can quantify copy numbers of methylated TWIS1 and methylated SEPT9 from 6.25 pg of control methylated DNA in a background of 5625 pg of control unmethylated DNA. We determined that an amount of 0.04 mL serum-derived DNA could be used as a template for digital PCR, in which the amount of DNA ranged from 104 to 9300 pg.

### CORD assay in serum samples

**Figure 3:**
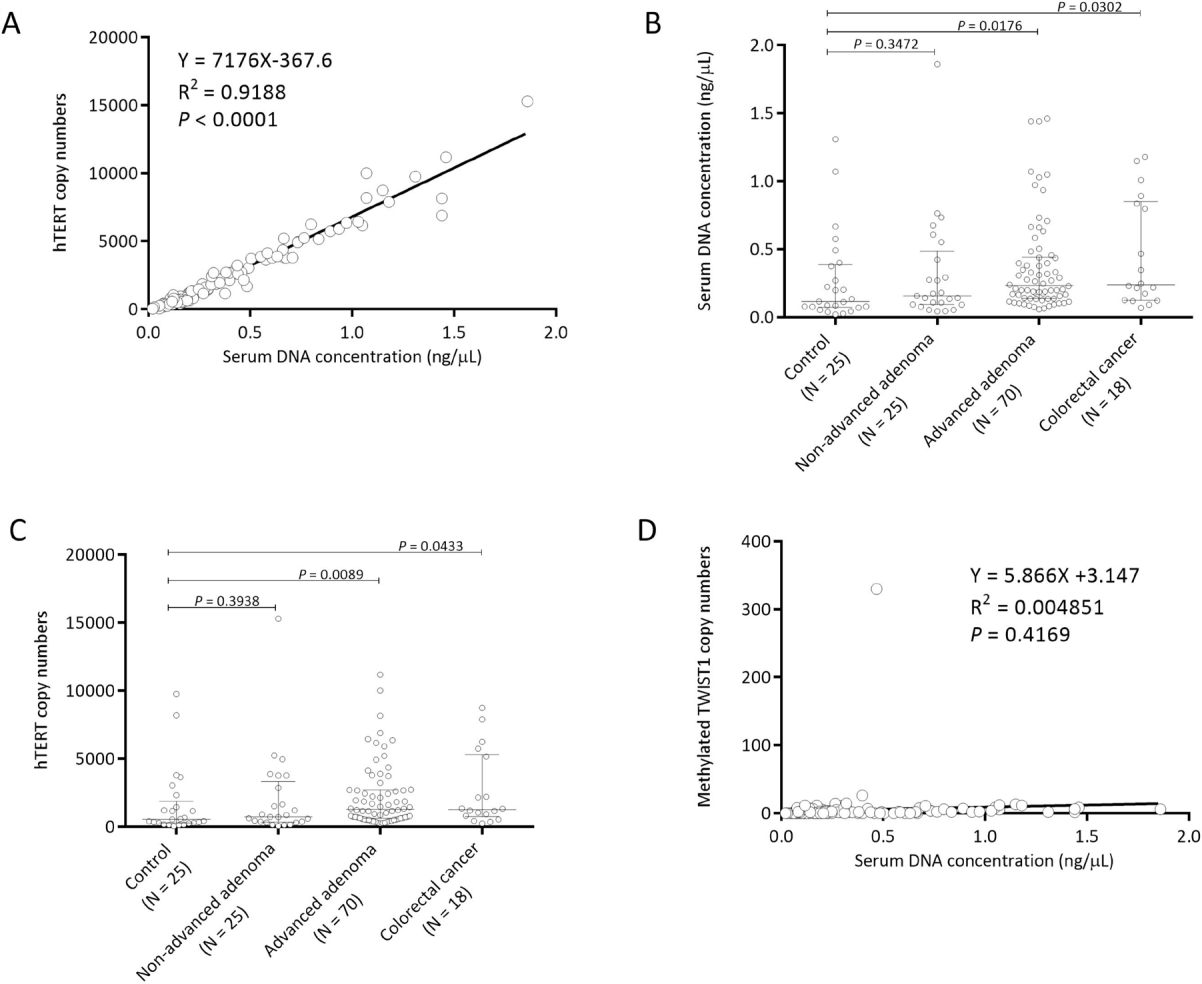
Serum DNA concentration is correlated with hTERT copy numbers but not with methylated TWIST1 copy numbers Correlations between serum DNA concentration (ng/μL in elution buffer) and copy numbers of TERT (**A**) and TWSIT1 (**D**) are shown. Distribution of serum DNA concentration (**B**) and hTERT copy number (**C**) in each group is shown. Each sample is indicated by an open circle. The copy numbers of hTERT and methylated TWSIT1 per an amount of DNA equivalent to the amount in 0.04 mL serum are shown. The box plots show the median with interquartile range (25th percentile and 75th percentile).

There was a linear relationship between DNA concentration and hTERT copy numbers (R^2^ = 0.9188, *P* < 0.0001; Figure 3A). Both serum DNA concentration and hTERT copy numbers were significantly higher in the advanced adenoma group and in the colorectal cancer group than in the control group (Figure 3B, 3C). The median serum DNA concentrations were 0.12, 0.16, 0.23, and 0.24 (ng/μL in elution buffer) (Figure 3B) and the median copy numbers of hTERT were 536 (range, 42 to 9760), 730 (range, 54 to 15300), 1274 (range, 234 to 11180), and 1258 (range, 244 to 8740) (Figure 3C) in the control group, non-advanced group, advanced group, and colorectal cancer group, respectively. In contrast, methylated TWIST1 copy numbers were independent of serum DNA concentration (Figure 3D).

**Figure 4:**
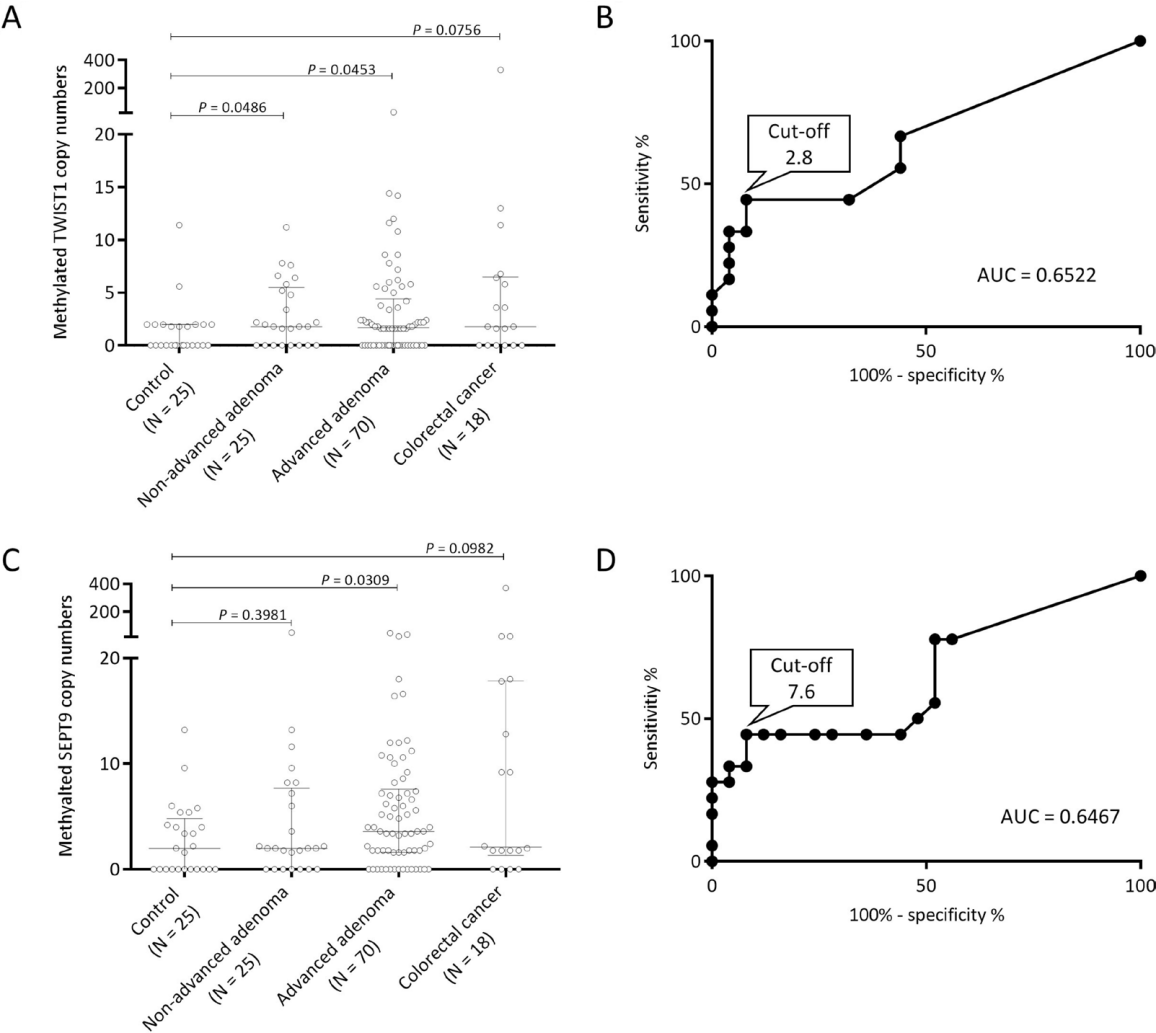
Distribution of methylation levels by serum CORD assay in each group Distribution of copy numbers of methylated TWIST1 (**A**) and methylated SEPT9 (**B**) in each group is shown. Each sample is indicated by an open circle. The copy numbers of TWIST1 and SEPT9 per an amount of DNA equivalent to the amount in 0.04 mL serum are shown. The box plots show the median with interquartile range (25th percentile and 75th percentile). Receiver-operating characteristic curve analysis of methylated TWIST1 (**C**) and methylated SEPT9 (**D**) to discriminate between the control group and CRC group is shown.

The median copy numbers of methylated TWIST1 were 0.0 (range, 0 to 11.4) in the control group, 1.9 (range, 0 to 11.2) in the non-advanced adenoma group, 1.7 (range, 0 to 26) in the advanced adenoma group, and 1.8 (range, 0 to 330) in the CRC group (Figure 4A). We performed receiver-operating characteristic (ROC) curve analysis to determine the best cutoff value to discriminate between the control group and CRC group. The area under the curve (AUC) was 0.6522, and we set 2.8 copies of methylated TWIST1 retrospectively as a tentative cut-off point (Figure 4B). The frequency above the cut-off point was 8.0% (2/25) of the individuals in the control group (specificity of 92%), 36.0% (9/25) in the non-advanced adenoma group, 30.0% (21/70) in the advanced adenoma group, 50.0% (7/14) in the stage I CRC group, and 44.4% (8/18) in the all-stages CRC group (Table 1 and Figure 1A). The false positive rate was 8.0% (2/25) for the control group, and the false-negative rates were 64.0% (16/25), 70.0% (49/70), 50.0% (7/14), and 55.6% (10/18) for the non-advanced adenoma, advanced adenoma, stage I CRC, and all-stages CRC groups, respectively. There was no association between TWIST1 methylation levels and clinicopathologic characteristics in the patients with colorectal neoplasia (Table 2).

**Table 2:** The association of TWIST1 gene methylation levels with clinicopathologic characteristics in patients with colorectal neoplasia

	Methylated TWIST1 (copies)		
	<2.8	≥2.8	*P*
Age in years (*N* = 113)			
Median (range)	67.0 (36–91)	68.5 (50–91)	0.3883
Sex (*N* = 113)			
Male	50	26	1.0000
Female	25	12	
Tumor location (*N* = 113)			
Right	44	23	1.0000
Left	31	15	
Tumor size (mm) (*N* = 113)			
Median (range)	20.0 (3–80)	19.0 (2–74)	0.4132
Tumor type (*N* = 113)			
Non-advanced adenoma	16	9	0.4918
Advanced adenoma	49	21	
Colorectal cancer	10	8	
Stage (*N* = 18)			
I	7	7	0.5968
II	1	0	
III	2	1	
Tumor differentiation (*N* = 18)			
Well	7	6	0.3910
Moderate	3	1	
Poor	0	1	

The median copy numbers of methylated SEPT9 were 2.0 (range, 0.0 to 13.2) in the control group, 2.0 (range, 0.0 to 50.0) in the non-advanced adenoma group, 3.6 (range, 0.0 to 46.0) in the advanced adenoma group, and 2.1 (range, 0.0 to 370.0) in the CRC group (Figure 4C). The best cutoff value to discriminate between the control group and CRC group was 7.6 copies of methylated SEPT9 by ROC curve analysis (Figure 4D). The frequency of individuals above the cut-off point was 8% (2/25) in the control group (specificity of 92%), 24.0% (6/25) in the non-advanced adenoma group, 24.3% (17/70) in the advanced adenoma group, 42.9% (6/14) in the stage I CRC group, and 44.4% (8/18) in the all-stages CRC group (Figure 1B). The false-positive rate was 8.0% (2/25) for the control group, and the false-negative rates were 76.0% (19/25), 75.7% (53/70), 57.1% (8/14), and 55.6% (10/18) for the non-advanced adenoma, advanced adenoma, stage I CRC, and all-stages CRC groups, respectively.

### Combination of FIT and serum CORD assay

The criterion for a positive result with the combination of FIT and CORD assay is either a positive FIT or CORD assay or both are positive. The combination of FIT and serum CORD assay of methylated TWIST1 resulted in a sensitivity of 40.0% (10/25) for non-advanced adenoma, 45.7% (32/70) for advanced adenoma, and 72.2% (13/18) for CRC, and the specificity was 84.0% (21/25) (Table 1 and Figure 1A). Focusing on stage I CRC, the combination test resulted in a sensitivity of 64.3% (9/14) (Figure 1A). The results of FIT and serum CORD assay of TWSIT1, in part, were mutually exclusive (Figure 1A). In the non-advanced adenoma group, 4.0% (1/25) of patients had a positive result of FIT alone, 32.0% (8/25) had a positive result of serum CORD assay alone, and only 4.0% (1/25) had positive results of both tests. In the advanced adenoma group, 15.7% (11/70) of patients had a positive result of FIT alone, 21.4% (15/70) had a positive result of serum CORD assay alone, and only 8.6% (6/70) had positive results of both tests. In the CRC group, 27.8% (5/18) of patients had a positive result of FIT alone, 27.8% (5/18) had a positive result of serum CORD assay alone, and only 16.7% (3/18) had positive results of both tests.

For methylated SEPT9, the combination of FIT and serum CORD assay resulted in a sensitivity of 28.0% (7/25) for non-advanced adenoma, 44.3% (31/70) for advanced adenoma, 42.9% (6/14) for stage I CRC, and 55.6% (10/18) for all-stages CRC, and the specificity was 80% (20/25) (Figure 1B).

## DISCUSSION

### Serum DNA concentration

In the present study, serum DNA concentration was significantly higher in the advanced adenoma group and CRC group than in the control group. Although increased amounts of circulating DNA in patients with CRC have been reported by other investigators [15, 16], an increase in the amount of circulating DNA in patients with advanced adenoma has not been reported. Therefore, this is the first report in the world, to our knowledge, to show that the amount serum DNA can be a promising biomarker of colorectal advanced adenoma. However, as an increase of the amount of blood free-circulating DNA is observed in various types of cancer [17, 18], serum DNA concentration would be a universal biomarker of various types of cancer and not limited solely to colorectal neoplasias.

### Serum CORD assay

The CORD assay can count copy numbers of a methylated target gene and an internal control gene, hTERT, simultaneously. We found that hTERT copy numbers correlated positively with serum DNA concentration and that they were significantly higher in the advanced adenoma group and the CRC group than in the control group. Thus, the hTERT copy number, as well as serum DNA concentration, may be a biomarker of colorectal advanced adenoma and CRC. As no investigators have reported serum hTERT copy number as a possible biomarker for detecting colorectal neoplasia, this may also be the first report to show that hTERT copy numbers can be a promising biomarker of colorectal neoplasia. However, as hTERT copy numbers are also increased in the plasma of patients with hepatocellular carcinoma [19, 20] and, as shown in the present study, there is a significant correlation between the serum hTERT copy number and serum DNA amount, serum hTERT copy number may also be a universal biomarker of various types of cancer and not limited only to colorectal neoplasias.

Regarding the methylation assay, although bisulfite treatment of DNA is commonly performed in the conventional methylation assays, this reaction introduces various DNA strand breaks and results in highly fragmented single-stranded DNA [11] and the loss of ~90% of the DNA [12]. The conventional bisulfite-based methylation assays require at least 10 copies of the target gene in the template DNA after bisulfite treatment of the DNA [21]. Thus, considering that ~90% of the DNA will be lost during bisulfite treatment [11], each template DNA must have at least 100 copies of the target gene prior to bisulfite treatment. The Epi proColon test, which is based on the conventional methylation assay, requires a large amount of plasma because PCR requires an amount of template DNA equivalent to the amount in approximately 0.9 mL plasma per well and is performed in triplicate. Thus, Epi proColon requires approximately 2.7 mL of plasma for a single test [8]. In contrast, in the serum CORD assay, an amount of template DNA equivalent to that in 0.04 mL of serum is enough because the CORD assay does not require bisulfite treatment of DNA. The methylation level is evaluated by droplet digital PCR, which allows the counting of even one copy of the target gene [22]. Thus, the cost of the CORD assay is lower, and the experimental technique is easier than those of the conventional methylation assays [4, 8, 13, 23].

Release of tumor markers including circulating tumor DNA occurs by vascular invasion into blood during the progression from pre-invasive polyps through the advancing stages of CRC [24], and the circulating tumor DNA possibly originates from apoptotic tumor cells, living tumor cells, and circulating tumor cells [25]. Interestingly, circulating epithelial cells in blood are detected in 10% of patients with colorectal adenoma and in 20% of patients with CRC [26]. Because we frequently observed hypermethylation of TWIST1 in the tissues of colorectal adenoma and cancer [14], we thought methylated TWIST1 would be available as a biomarker of blood-based DNA testing for the detection of colorectal neoplasia including adenoma and cancer. In the present study, the serum CORD assay of methylated TWIST1 resulted in a sensitivity of 36.0%, 30.0%, and 44.4% for the detection of non-advanced adenoma, advanced adenoma, and CRC, respectively, showing better performance compared with CEA (16.7%, 16.7%, and 17.6%, respectively) and equal or better performance compared with FIT (8.0%, 24.3%, and 44.4%, respectively). Regarding specificity, three individuals in the control group had positive (false-positive) results by FIT, whereas only two individuals in the control group had positive results by CORD assay of methylated TWIST1, suggesting that the CORD assay of methylated TWIST1 might have higher specificity than FIT.

In the current study we also established serum CORD assay of SEPT9 because Epi proColon is not available in Japan. Compared to SEPT9, the serum CORD assay of TWIST1 showed better performance for colorectal neoplasia screening, especially for non-advanced adenoma. In addition, the serum CORD assay of methylated TWIST1 showed moderate or better sensitivity as compared with those in previous reports of blood-based testing of methylated SEPT9 including the Epi proColon, in which the sensitivity is 7% for non-advanced adenoma, 11% for advanced adenoma, 48%-68% for CRC (35%–41% for CRC stage I, 63%–83% for CRC stage II, 46%–80 for CRC stage III, and 77%–100% for CRC stage IV) [27, 28]. The 92% specificity of the serum CORD assay of methylated TWSIT1 was non-inferior to that of the 73%–93% achieved with blood testing of methylated SEPT9 [29]. Because the sample size in this study is small, further studies with larger sample sizes are required to determine the best cut-off point of the TWIST1 methylation level and to confirm the diagnostic accuracy of the CORD assay for colorectal neoplasia screening.

### Combination of FIT and serum CORD assay of methylated TWIST1

The sensitivity of FIT for the detection of non-advanced adenoma and advanced adenoma is, in general, quite low [4] because the amount of bleeding in stool from these tumor types is too small to be detected by fecal occult blood tests [30, 31]. However, the serum CORD assay seemed to complement FIT as a different screening modality for these tumor types because the results of FIT and serum CORD assay of methylated TWIST1, in part, seemed mutually exclusive. Therefore, we thought the combination of serum CORD assay of methylated TWIST1 and FIT might improve the sensitivity as compared to that by FIT alone. Indeed, this combination resulted in a sensitivity of 40.0%, 45.7%, and 72.2% for non-advanced adenoma, advanced adenoma, and CRC detection, respectively, showing higher sensitivities than those by FIT alone (8.0%, 24.3%, and 44.4%, respectively). Furthermore, the combination of the two increased the sensitivity to 64.3% for stage I CRC detection, whereas the sensitivity of FIT alone was only 28.6%. Although the disadvantage of a multi-marker test, in general, is a decrease in specificity [4], there was no great difference in the specificity between the combination test (serum CORD assay of methylated TWIST1 and FIT) and FIT alone (84% versus 88%) in the present study. In addition, the combination of serum CORD assay of methylated TWIST1 and FIT showed better sensitivity and specificity than that of serum CORD assay of methylated SEPT9 and FIT. Thus, the combination test consisting of serum CORD assay of methylated TWIST1 and FIT may be useful as a laboratory test for the detection of early-stage CRC and non-advanced and advanced adenoma. Because of the small sample size and the bias in CRC patients for stage I (14/18), further studies with larger sample sizes including advanced CRC are required to confirm the usefulness of the combination of serum CORD assay and FIT for colorectal neoplasia screening.

It is reported that resection of adenomatous polyps of the colon and rectum by colonoscopic polypectomy reduces the incidence of CRC by 76–90% [32] and prevents death from CRC, with a 53% reduction in mortality [33]. Thus, screening of adenomatous polyps including non-advanced and advanced adenoma by combination of serum CORD assay and FIT is expected to motivate individuals to undergo colonoscopy and to lead to reductions in the incidence and mortality of CRC. Further investigation will be required to prove this hypothesis.

In this study, detection of methylated TWIST1 from serum samples appeared to be useful for colorectal neoplasia screening. However, we admit that hypermethylation of TWIST1 is associated with different types of cancer: breast, uterine cervix, ovary, bladder, gastric, lung, bone, pancreas, and brain [34–44]. Thus, an increase in the methylated TWIST1 level in blood may suggest the presence of some kind of cancer, not just that limited to colorectal neoplasia. Indeed, in the current study, two participants in the control group had high methylated TWIST1 copy numbers. Both subjects had no previous history of any types of cancer. One subject was a man in his sixties and a former-smoker with 18 pack-years. The other was a woman in her fifties and a current smoker with 15 pack-years. As tobacco use causes many types of cancer [45], a medical check-up and a follow-up survey will be required for these subjects. To clarify the usefulness of DNA testing of methylated TWIST1 as a universal tumor marker from blood samples, retrospective and prospective cohort studies comprising various types of cancer are required. Compared to other methylation assays, the CORD assay has a great advantage especially in performing retrospective studies using biobank resources because the amount of blood samples commercially available from biobanks is usually less than 1 mL [46]. In the conventional methylation assays, a large amount (up to 4 mL) of plasma or serum is required [8, 23, 47]. In contrast, in the CORD assay, an amount of DNA equivalent to that in 0.04 mL serum is enough for a single test. Thus, the CORD assay will easily enable the measurement of methylation levels of various genes and can confirm the reproducibility of data from archived blood samples in a biobank.

In conclusion, the combination of TWIST1 methylation analysis by serum CORD assay and FIT showed higher sensitivities for the detection of non-advanced adenoma, advanced adenoma, and early-stage CRC without great difference in the specificity as compared to those by FIT alone. Because this study suggests that the combination of serum DNA testing of methylated TWIST1 and FIT may be useful to detect individuals with colorectal neoplasia, confirmatory studies using independent data sets are needed to support our findings.

## MATERIALS AND METHODS

### Materials

**Figure 5:**
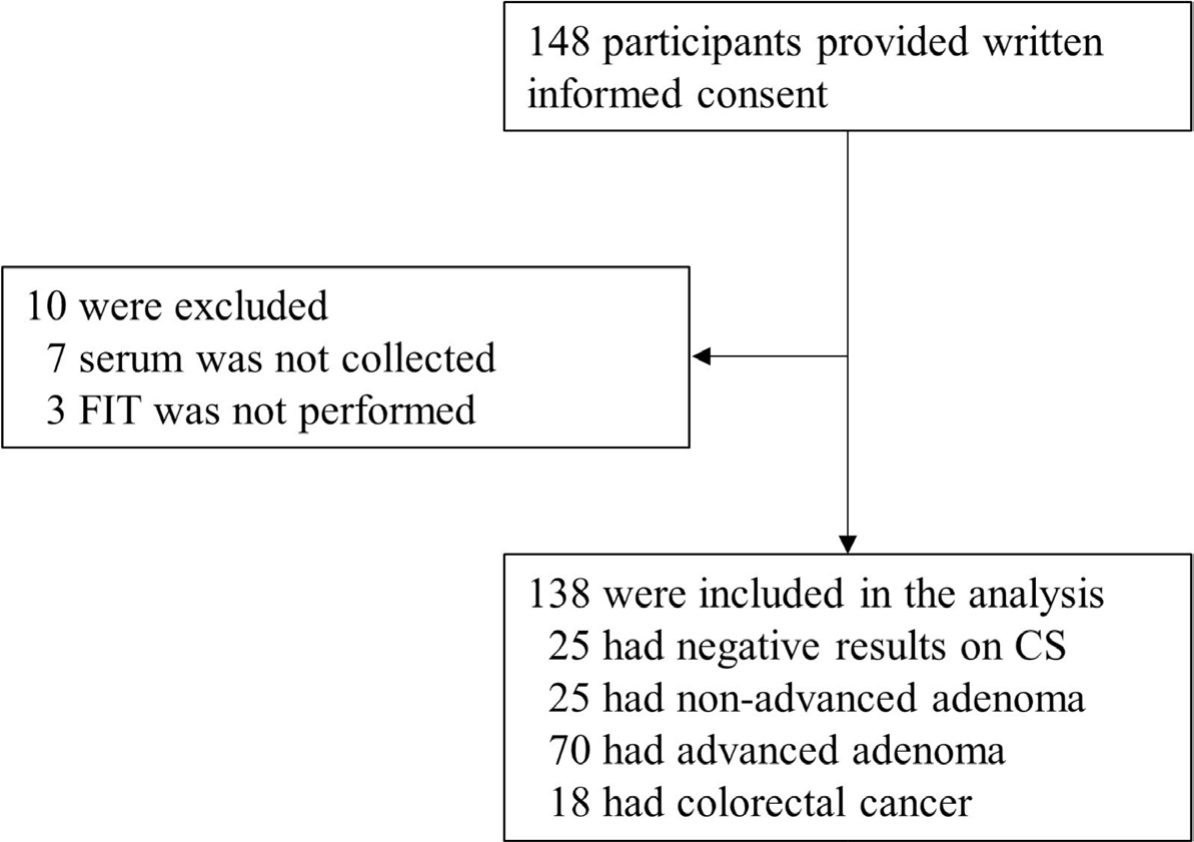
Enrollment and outcomes CS: colonoscopy; FIT: fecal immunochemical test.

We enrolled 148 participants of whom 138 had results that could be fully evaluated (Figure 5). Serum was prospectively collected in advance of bowel preparation for colonoscopy between March 15, 2015 and April 8, 2017 in Yamaguchi University Hospital, Sentohiru Hospital, or Ajisu Kyoritsu Hospital and stored at −80°C until DNA extraction. The subjects comprised 25 healthy volunteers without colorectal neoplasia as determined by colonoscopy (control group), 25 patients with colorectal non-advanced adenomas (non-advanced adenoma group), 70 patients with advanced colorectal adenoma (advanced adenoma group), and 18 patients with CRC (cancer group) diagnosed by endoscopic or surgical resection. All subjects were Japanese. We advertised via flyers and posters for healthy volunteers who were able and willing to undergo screening colonoscopy, had never undergone colonoscopy, had had no medical history of any cancers or inflammatory bowel diseases, and had had no positive FIT within the previous one year. We clarified the purpose of this study and complications and risks of colonoscopy in face-to-face interviews using verbal explanation and the consent form. The healthy volunteers were limited to those who participated spontaneously, understood the risk of colonoscopy, and signed a written informed consent document. Adenomas of less than 1 cm in size with no high-grade dysplasia or villous component were categorized as non-advanced adenoma [4]. Criteria of advanced adenoma were defined as adenomas of 1 cm or greater in size, or with villous components (tubulovillous or villous), or with high-grade or severe dysplasia [4]. Staging was classified according to the International Union Against Cancer (UICC) [48]. Clinicopathologic characteristics of the participants are shown in Table 3. The study protocol was approved by the institutional review boards of Yamaguchi University Graduate School of Medicine, Sentohiru Hospital, and Ajisu Kyoritsu Hospital, and informed consent was obtained from each patient and each healthy volunteer.

**Table 3:** Clinicopathologic characteristics

	Carcinoma (*N* = 18)	Advanced adenoma (*N* = 70)	Non-advanced adenoma (*N* =25)	Control (*N* = 25)
Age in years				
Median (range)	71.0 (41–91)	67.5 (36–91)	66.0 (37–81)	55.0 (33–79)
Sex				
Male	13	48	15	10
Female	5	22	10	15
Tumor location				
Right	10	46	11	
Left	8	24	14	
Tumor size (mm)				
Median (range)	26 (6–60)	20 (5–80)	5 (2–8)	
pStage				
I	14			
II	1			
III	3			
Tumor differentiation				
Well	13			
Moderate	4			
Poor	1			
Copy numbers (median)				
Methylated TWIST1	1.8	1.7	1.9	0.0
Methylated SEPT9	2.1	3.6	2.0	2.0
hTERT	1258	1274	730	536
Serum DNA concentration				
Median (ng/mL)	0.24	0.23	0.16	0.12

### Fecal immunochemical test for hemoglobin

Participants received illustrated Japanese-language instructions on sampling feces from one bowel movement by briefly sweeping the tip of a probe several times though the feces [49]. Fresh fecal specimens were collected into the sampling containers filled with 2 mL of a hemoglobin-stabilizing buffer solution (Eiken Kagaku, Tokyo, Japan) prior to bowel preparation for the colonoscopy procedure [50]. FIT was performed using OC-HEMODIA (Eiken Kagaku, Tokyo, Japan), a latex agglutination FIT with analytical characteristics. The OC-Sensor IO instrument processed and quantified the FIT results at the manufacturer-recommended concentration cut-off value of 20 μg hemoglobin/g feces units (100 ng hemoglobin/1 mL of buffer) for a positive test result [50].

### CEA

Serum carcinoembryonic antigen (CEA) was measured in 76 patients with colorectal neoplasms using the “TOSOH” II CEA commercial immunoassay kit (Tosoh Corporation, Tokyo, Japan) and an AIA-2000 automatic immunoassay analyzer (Tosoh Corporation) in the laboratory division of Yamaguchi University Hospital. The cutoff value of the serum CEA levels was set at 6 ng/mL following the manufacturer's instruction.

### Sample preparation and DNA extraction

DNA from peripheral blood leukocytes was used as a control for unmethylated TWIST1 and unmethylated SEPT9, and DNA from CRC cell line HCT116 was used as a control for hypermethylated TWIST1 [14] and hypermethylated SEPT9 [51]. Serum samples were thawed from −80°C, and 0.4 mL of each sample was used for DNA extraction with the MagNA Pure Compact Nucleic Acid Isolation Kit I (Roche, Tokyo, Japan) according to the manufacturer's instructions. DNA was eluted in a volume of 50 μL of elution buffer and quantified using Qubit 2.0 fluorometers (Thermo Fisher Scientific, Yokohama, Japan).

### CORD assay

We performed CORD assay consisting of two-step treatments of DNA with multiple methylation-sensitive restriction enzymes followed by multiplex digital PCR [13]. In the first step of enzyme treatment, 10 μL of eluted DNA (an amount of DNA equivalent to that in 80 μL serum) was digested for 16 hours at 37°C by the addition of 1 μL of GeneAmp 10x PCR Buffer II, 1 μL of 25 mmol/l MgCl_2_, 10 units of Hha I, 10 units of Hpa II, and 20 units of exonuclease I (Exo I) (all from Thermo Fisher Scientific). Exo I was added to eliminate single-stranded DNA that would escape digestion by the restriction enzymes and to avoid PCR amplification of the undigested fraction [52]. In the second step, additional digestion of DNA was performed for 16 hours at 60°C using 10 units of BstUI (New England Biolabs Ltd., Hitchin, UK). After the restriction was complete, the mixture was heated for 10 min at 98°C. The TWIST1 had six recognition sites of methylation-sensitive enzymes HhaI, HpaII, and BstUI. When all six of the sites were methylated, the target DNA would escape digestion by these enzymes and would be amplified by PCR. Similarly, the SEPT9 had three recognition sites of methylation-sensitive enzymes HhaI and BstUI. When all three of the sites were methylated, the target DNA would escape digestion by these enzymes and would be amplified by PCR. Regarding hTERT, as no recognition sites of theses enzymes existed in the target sequences, hTERT was always amplified by PCR when human DNA was present in the template DNA.

We performed multiplex droplet digital PCR to count the absolute copy numbers of hTERT, methylated TWIST1, and methylated SEPT9. The PCR reaction solution consisted of 8 μL of enzyme-treated DNA (an amount of DNA equivalent to that in 0.04 mL serum), 1 × ddPCR Supermix for Probes (Bio Rad, Tokyo, Japan), 0.25 μmol/L of each primer of a target gene and an internal control, and 0.125 μmol/L of each probe of a target gene and an internal control in a total volume of 20 μL. The sequences of the primer and probe set of TWIST1 were as follows: forward primer, 5′-TCCAAAGGCCAAACCGC-3′; reverse primer, 5′-CCGGGACGCAAATCCTC-3′; probe, 5′-FAM-CTGAAGACGTGGCCGCGCC-TMARA-3′. The PCR amplicon length is 92 bp from 19,157,854 to 19,157,945 of chromosome 7 (human assembly GRCh37/hg19). Those for hTERT were forward primer, 5′-GGGTCCTCGCCTGTGTACAG-3′; reverse primer, 5′-CCTGGGAGCTCTGGGAATTT-3′; probe, 5′-VIC- CACACCTTTGGTCACTC-MGB-3′ [53]. The PCR amplicon length is 60 bp from 1,253,375 to 1,253,434 of chromosome 5 (human assembly GRCh37/hg19). We designed SEPT9 primers and probes within the CpG island 3 region of SEPT9 containing the transcription start site of SEPT9 transcript variant 2, which was previously described as differentially methylated in colorectal cancer and the target of Epi proColon [54, 55]. The sequences of the primer and probe set of SEPT9 were as follows: forward primer, 5′- GCCCACCAGCCATCATGT-3′; reverse primer, 5′- GTCCGAAATGATCCCATCCA-3′; probe, 5′-FAM- CCGCGGTCAACGC-MGB-3′. The PCR amplicon length is 62 bp from 75,369,566-75,369,627 of chromosome 17 (human assembly GRCh37/hg19). Droplet generation was performed by an automated droplet generator (Bio Rad) and was followed by PCR. PCR cycling conditions included preheating at 95°C for 10 min followed by 40 cycles of denaturation at 94°C for 30 s, annealing at 56°C for 60 s, and final heating at 98°C for 10 min. After amplification, the PCR plate was transferred to a QX100 droplet reader (BioRad), and fluorescence amplitude data were obtained by QuantaSoft software (BioRad).

### Statistical analyses

To compare variables, the Mann–Whitney *U* test, chi-square test, Fisher's test, and linear regression analyses were used. A *P* value of less than 0.05 was considered statistically significant. Statistical analyses were performed with GraphPad InStat Ver. 3, and GraphPad Prism Ver. 6 statistical software (GraphPad Software, La Jolla, CA).

## Abbreviations

CEA: carcinoembryonic antigen
CORD: combined restriction digital PCR
CRC: colorectal cancer
FIT: fecal immunochemical test for hemoglobin
